# Antagonistic Cross-Regulation between Sox9 and Sox10 Controls an Anti-tumorigenic Program in Melanoma

**DOI:** 10.1371/journal.pgen.1004877

**Published:** 2015-01-28

**Authors:** Olga Shakhova, Phil Cheng, Pravin J. Mishra, Daniel Zingg, Simon M. Schaefer, Julien Debbache, Jessica Häusel, Claudia Matter, Theresa Guo, Sean Davis, Paul Meltzer, Daniela Mihic-Probst, Holger Moch, Michael Wegner, Glenn Merlino, Mitchell P. Levesque, Reinhard Dummer, Raffaella Santoro, Paolo Cinelli, Lukas Sommer

**Affiliations:** 1 Cell and Developmental Biology, Institute of Anatomy, University of Zurich, Zurich, Switzerland; 2 Department of Dermatology, University Hospital Zurich, Zurich, Switzerland; 3 Laboratory of Cancer Biology and Genetics, National Cancer Institute, Bethesda, Maryland, United States of America; 4 Department of Pathology, Institute of Surgical Pathology, University Hospital Zurich, Zurich, Switzerland; 5 Institute of Biochemistry, Emil Fischer Center, FAU University of Erlangen-Nuernberg, Erlangen, Germany; 6 Institute of Veterinary Biochemistry and Molecular Biology, University of Zurich, Zurich, Switzerland; 7 Division of Trauma Surgery, Center for Clinical Research, University Hospital Zurich, Zurich, Switzerland; 8 Department of Oncology, University Hospital Zurich, Schlieren, Switzerland; Stanford University School of Medicine, United States of America

## Abstract

Melanoma is the most fatal skin cancer, but the etiology of this devastating disease is still poorly understood. Recently, the transcription factor Sox10 has been shown to promote both melanoma initiation and progression. Reducing SOX10 expression levels in human melanoma cells and in a genetic melanoma mouse model, efficiently abolishes tumorigenesis by inducing cell cycle exit and apoptosis. Here, we show that this anti-tumorigenic effect functionally involves SOX9, a factor related to SOX10 and upregulated in melanoma cells upon loss of SOX10. Unlike SOX10, SOX9 is not required for normal melanocyte stem cell function, the formation of hyperplastic lesions, and melanoma initiation. To the contrary, SOX9 overexpression results in cell cycle arrest, apoptosis, and a gene expression profile shared by melanoma cells with reduced SOX10 expression. Moreover, SOX9 binds to the SOX10 promoter and induces downregulation of SOX10 expression, revealing a feedback loop reinforcing the SOX10 low/SOX9 high ant,m/ii-tumorigenic program. Finally, SOX9 is required *in vitro* and *in vivo* for the anti-tumorigenic effect achieved by reducing SOX10 expression. Thus, SOX10 and SOX9 are functionally antagonistic regulators of melanoma development.

## Author Summary

For the development of future cancer therapies it is imperative to understand the molecular processes underlying tumor initiation and expansion. Many key factors involved in these processes have been identified based on cell culture and transplantation experiments, but their relevance for tumor formation and disease progression in the living organism is often unclear. Therefore, genetically modified mice spontaneously developing tumors present indispensable models for cancer research. Here, we address this issue by studying the formation of melanoma, the most fatal skin tumor in industrialized countries. To this end, we use a transgenic mouse model to elucidate cellular and molecular mechanisms regulating congenital nevus and melanoma initiation. We show that a transcription factor called SOX10 promotes melanoma formation by repressing an anti-tumorigenic program involving the activity of a related factor, SOX9. When SOX10 is inactivated, SOX9 becomes upregulated and induces cell cycle arrest and death in melanoma cells. Furthermore, upon experimental elevation of SOX9 levels, SOX10 activity is suppressed, revealing an antagonistic relationship between SOX9 and SOX10 in melanoma initiation. Knowledge of how an anti-tumorigenic program can be stimulated by modulating the activities of these key factors might help to design novel therapeutic strategies.

## Introduction

*Sox* (Sry (sex determining region Y)-related HMG box) genes encode a family of transcription factors that are characterized by a conserved high-mobility group (HMG) domain mediating their binding to DNA in a sequence-specific manner [1–3]. While the majority of Sox proteins functions as transcriptional activators, some members of the Sox family including Sox9 and Sox10 may also act as transcriptional repressors [4–6]. *Sox* genes play key roles in embryonic development and are major determinants of stem cell behavior, regulating cell fate decisions and maintaining cellular identity [3]. Their crucial role in normal tissue formation and homeostasis is evident from the fact that several mutations in Sox genes are causative for developmental diseases, and accumulating evidence demonstrates the important functional role of Sox family proteins in a variety of cancers [7–10].

A common feature of SoxE group proteins, which includes Sox9 and Sox10, is their expression in neural crest (NC) cells during embryonic development [2, 11]. NC cells are a transient embryonic cell population that gives rise to most of the peripheral nervous system, chondrocytes and osteoblasts of craniofacial structures, smooth muscle cells of the cardiovascular system, and melanocytes, the pigmented cells of the skin [12]. While Sox9 is expressed in premigratory NC cells and in the pharyngeal apparatus, Sox10 is found in NC cells at the time of their emigration and is essential for their self-renewal and survival [12–16]. Loss of *Sox10* results in absence of most NC derivatives, whereas *Sox10* haploinsufficiency causes Waardenburg Hirschsprung syndrome, characterized by aganglionic megacolon, pigmentary abnormalities and often deafness due to loss of sensory innervation [13, 17–20]. In the melanocytic lineage, Sox10 is expressed during all stages of development as well as in the adult and is required in different species for the generation and homeostasis of embryonic and adult melanocytes *in vitro* and *in vivo* [13, 21–25].

In contrast, loss of Sox9 in the NC does not lead to general defects in NC-derived structures, but specifically affects the development of mesectodermal derivatives, such as smooth muscle cells and craniofacial bones and cartilage [11, 26–28]. Furthermore, heterozygous mutations in *Sox9* in both mice and humans, result in campomelic dysplasia, a syndrome associated with dwarfism, skeletal malformations, cleft palate, XY sex reversal and often hermaphroditism [28–30]. However, data on Sox9 expression in melanocytes are inconsistent, and a functional implication of Sox9 in melanocyte formation has not been provided so far [23].

Based on the assumption that mechanisms of tumor formation might be related to those underlying the generation of the cell type, from which the tumor develops, we and others have recently addressed the function of Sox10 in melanoma. These studies demonstrated a crucial role of Sox10 in the pathogenesis of giant congenital naevi and melanoma in both mice and humans by regulating proliferation and survival of melanocytic cells and maintenance of their cellular identity [9; 31]. However, the precise molecular mechanisms mediating Sox10 function in melanoma remain to be investigated.

Here we show that in contrast to *Sox10, Sox9* appears to be expressed at very low levels only and is functionally not required in melanocyte stem cells, committed melanocytes, and melanoma cells. However, Sox9 expression is elevated upon *Sox10* deletion in mouse and human melanoma cells [9], and critically contributes to the anti-tumorigenic effects observed upon *Sox10* inactivation in giant congenital naevus and melanoma.

## Results

### Expression analysis of SOXE factors in human skin melanocytes, giant congenital naevi, and primary melanoma *in vivo*

While SOX10 expression and function has been well established in adult melanocytes, naevi, and melanoma tissue in human and mice [9; 23], studies on SOX9 expression in melanocytic cells are controversial. SOX9 was reported to be expressed in cultured human melanocytes *in vitro* [32], human melanocytes *in vivo* [33], and in human melanoma [34–36]. Other reports, however, failed to reveal Sox9 mRNA and protein expression in melanoblasts and differentiated melanocytes during development and postnatally [21,37]. Given the close relationship between SoxE factors, one conceivable explanation for these discrepancies might be that antibodies raised against a given SoxE protein fail to discriminate between SOX9 and SOX10 epitopes. Indeed, when we performed immunohistochemistry on murine skin to test the specificity of various anti-SOX9 antibodies, several of them recognized both melanocytes and epithelial cells in the outer root sheet (a region in hair follicles known to express and functionally require Sox9; [37–38] (S1 Fig.). In contrast, the antibody sc-20095 exclusively detected protein expression in epithelial cells but not in melanocytes. Of note, in human melanoma cell lines *in vitro*, all antibodies tested but sc-20095 not only recognized SOX9, but also a protein of the molecular weight of SOX10 and detected by a SOX10-specific antibody (S2A-S2D Fig.).

To further investigate the specificity of anti-SOX9 antibodies, we performed SOX10 knockdown in human melanoma cell lines *in vitro* and analyzed SOX10 and SOX9 expression using Western blot analysis (S2E-S2K Fig.). As shown in S2E-S2K Fig., different anti-SOX9 antibodies used in earlier studies detected SOX10 protein expression, which was lost upon SOX10 knock-down. The only anti-SOX9 antibody, which did not display cross-reactivity with SOX10 protein, was sc-20095.

**Figure 1:**
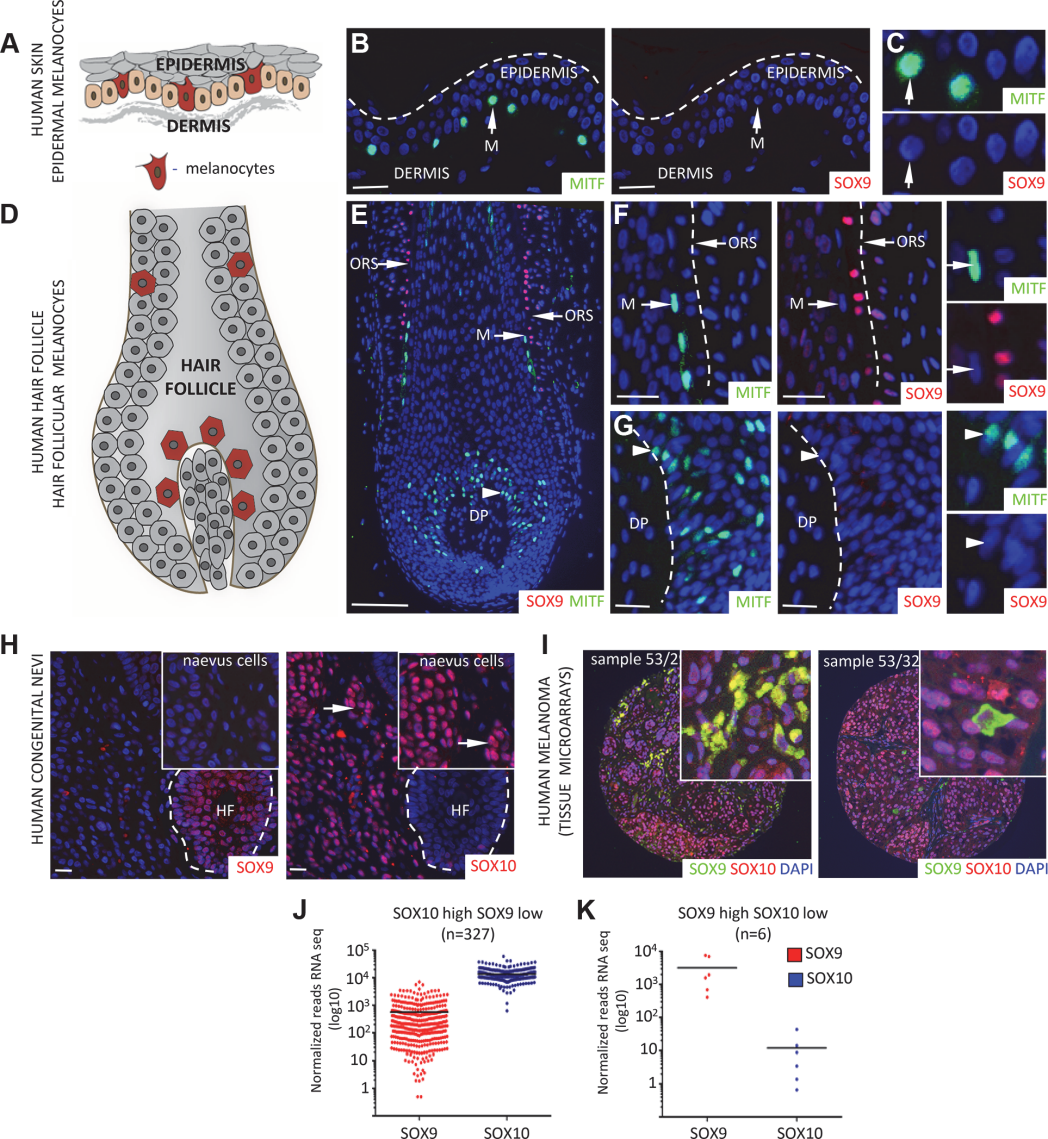
Differential expression of SOX10 and SOX9 in human melanocytes, human giant congenital naevi and human melanoma samples. **A**, Scheme showing the localization of epidermal melanocytes (in red) in the human skin. **B, C**, Immunostaining for MITF (green, left panel) and SOX9 (red, right panel) in the human skin demonstrating the lack of SOX9 expression in the epidermal melanocytes. Inserts show higher magnification images of MITF and SOX9 immunostainings. Scale bars, 25 μm. **D**, Scheme showing the localization of melanocytes (in red) within the hair follicle. **E**, Immunostaining for MITF (green) and SOX9 (red) in the human skin reveals the expression of SOX9 in the cells of outer root sheath but not in the MITF-positive melanoblasts/melanocytes. Scale bar 100 μm. **F, G**, High magnification images of immunostaining for MITF and SOX9 in the upper part of human hair follicle (**F**) and the follicular bulb (**G**). **H**, Analysis of SOX9 (red, left panel) and SOX10 (red, right panel) expression in the patients with human giant congenital naevi demonstrates the lack of SOX9 expression in the SOX10-positive giant congenital naevi cells. Inserts show higher magnification. **I**, Representative examples of immunostaining for SOX9 (green) and SOX10 (red) in a tissue microarray of primary melanoma samples are shown. **J-K**, Distribution of SOX10 vs. SOX9 expression in human melanoma (based on TCGA database). 334 melanoma patients were divided in two groups, namely SOX10 High/ SOX9 Low and SOX10 Low / SOX9 high based on SOX10 and SOX9 expression levels. DP, dermal papilla; HF, hair follicle; M, melanocytes; ORS, outer root sheath. Scale bars, 25 μm.

Therefore, we chose to reassess SOX9 expression in human melanocytes and melanocytic skin lesions using the specific SOX9 antibody sc-20095. Double immunostaining for SOX9 and MITF (Microphthalmia-associated transcription factor), an established marker of melanocytes [39] revealed no detectable SOX9 expression in human skin melanocytes *in vivo* (Fig. 1A-G). In contrast, SOX10 was readily detectable in human melanocytes (S3A-S3B Fig.). Moreover, while SOX10 was expressed in 100% of human giant congenital naevi, SOX9 expression was not detected in the same set of patient samples (n = 17; (Fig. 1H; S3D Fig.); [9]). Likewise, all samples of a melanoma tissue microarray composed of 56 primary melanoma biopsies revealed strong SOX10 expression (Fig. 1I; [9]). SOX9 expression, however, was found in only 41% (23/56) of the primary melanoma samples, in which it was expressed in a few scattered cells accounting for less than 10% of all melanoma cells (Fig. 1I). In contrast, expression of SOX9 was readily detectable in the epithelial lineage of normal skin as well as in basal cell carcinoma, an epithelial skin cancer (Fig. 1E, F; S3C Fig.; [37,40]). To investigate the mRNA expression of SOX10 and SOX9 in a large set of human melanoma samples, we used of the TCGA (The Cancer Genome Atlas) database. Interestingly, the vast majority of human melanoma samples displayed much higher SOX10 than SOX9 expression (Fig. 1J) and only very few samples were characterized by a SOX9 high / SOX10 low expression pattern (Fig. 1K). Thus, SOX10 but not SOX9 is prominently expressed in normal human melanocytes, human giant congenital naevi, and primary melanoma.

### SOX10 and SOX9 exhibit divergent functional roles in murine melanocyte stem cells and hair pigmentation

**Figure 2:**
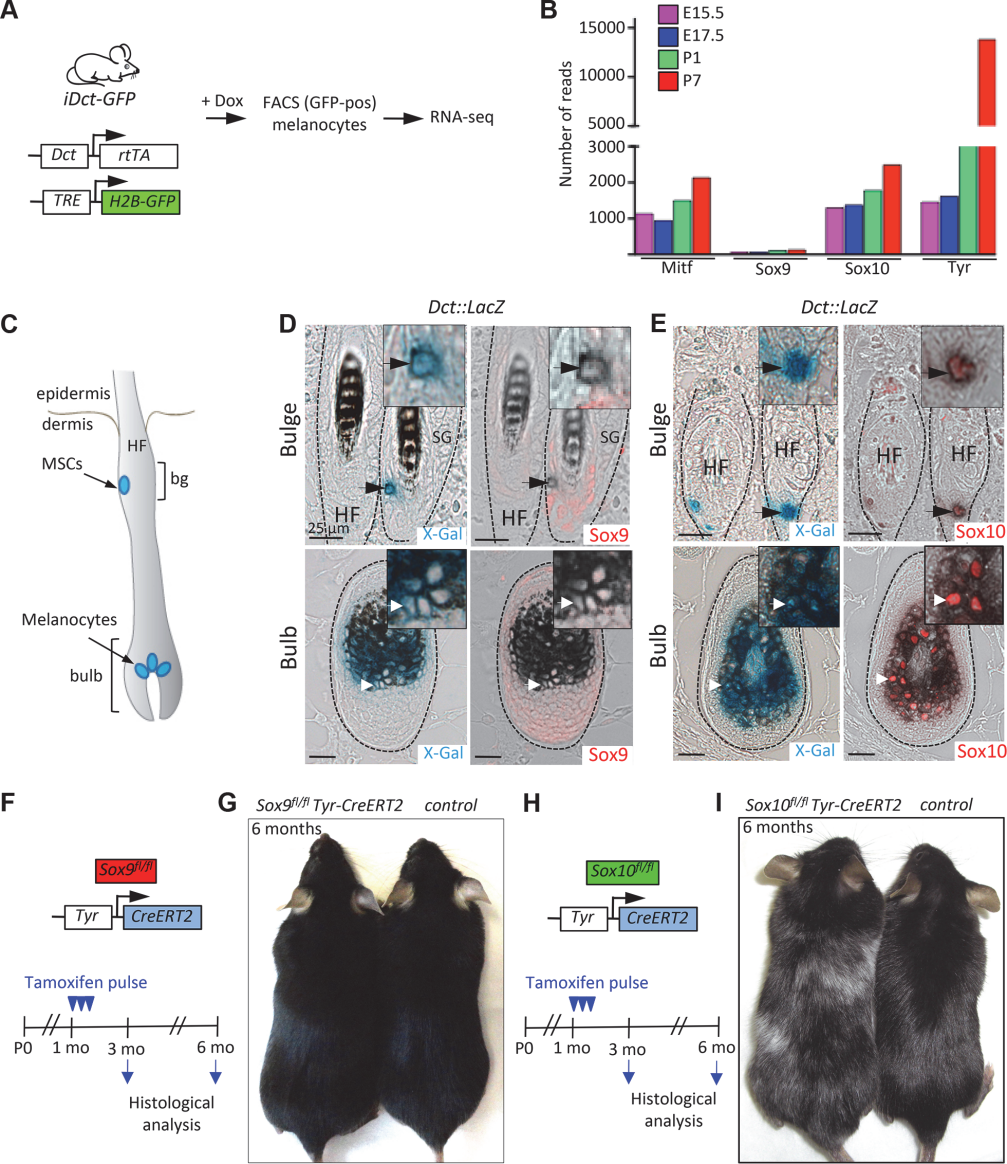
Sox9 is not expressed in the mouse melanocytic lineage and is not functionally required for the maintenance of melanocyte stem cells and melanocytes in the postnatal skin. **A**, A schematic representation of the experimental strategy used to analyze the expression of *Sox9, Sox10, Mitf* and *Tyr* genes in the melanocytic lineage *in vivo*. **B**, Results of RNA-seq analysis demonstrating high Sox10 and low Sox9 expression in melanocytic cells at various stages of development. **C**, A schematic representation of the anatomical location of the melanocyte stem cells, melanoblasts and differentiated melanocytes within the hair follicle in the mouse skin. **D**, X-Gal staining (blue) combined with Sox9 immunostaining (red) in skin sections of *Dct::LacZ* mice demonstrating the lack of the Sox9 expression in the melanocyte stem cells located in the bulge region of the hair follicle (upper panels) and in the differentiated melanocytes located in the hair follicular bulb (lower panels). **E**, Skin sections of *Dct::LacZ* mice stained for Sox10 (red) in combination with X-Gal staining (blue) reveal the expression of Sox10 in the melanocyte stem cells (upper panels) as well as in the differentiated melanocytes (lower panels). Dashed lines demarcate HFs. Insets show high magnification views. **F-I**, Experimental strategy used to analyze the effect of the lack of *Sox9* (**F**) and *Sox10* (**H**) expression in the mouse melanocytic lineage. Pictures of two representative mice at 6 months of age lacking *Sox9* gene (**G**) and *Sox10* gene (**I**) demonstrating the effects on hair graying. Bg, bulge; HF, hair follicle; MSCs, melanocyte stem cells; Mo, months; E 15.5, embryonic day 15.5; P0, postnatal day 0; SG, sebaceous gland. Scale bars, 25 μm.

To corroborate our findings in an experimentally amenable system, we extended the analysis of Sox9 expression to mouse melanocytes, taking advantage of a previously described iDct-GFP mouse line (Fig. 2A,B; [41]). Doxycycline-induced GFP-labelled melanocytes were isolated via fluorescence-activated cell sorting (FACS) and subjected to RNA-Seq analysis (Fig. 2A). While Sox10, Mitf and Tyr were expressed at high levels (Sox10 reads were 1292, 1372, 1776 and 2488 at E15.5, E17.5, P1 and P7, respectively), the expression of Sox9 was extremely low (Sox9 reads were 68, 65, 105 and 128 at E15.5, E17.5, P1 and P7, respectively). These data are in accordance with earlier studies on Sox9 mRNA and protein expression in murine melanoblasts and melanocytes [21,37] and suggest that in contrast to Sox10, Sox9 expression is virtually absent in the melanocytic lineage during mouse embryogenesis and postnatally.

Melanocyte stem cells are found in a specialized region of the hair follicle called bulge and give rise to melanocyte progenitors and differentiated melanocytes [42]. The latter are located in the lower hair follicle portion termed bulb, where they transfer pigment to the growing hair. When melanocyte stem cells are functionally impaired, they fail to generate melanocytes, which results in hair graying [43]. To further investigate the expression of Sox10 and Sox9 in the melanocytic lineage of the mouse skin, we made use of *Dct::LacZ* transgenic mouse line expressing LacZ driven by the dopachrome tautomerase (Dct) promoter that allows genetic tracking of melanocyte stem cells and their derivatives in the hair follicle (Fig. 2C; [44]). Sox10 expression was detected in X-Gal-positive melanocyte stem cells located in the bulge region (Fig. 2E, upper panels) as well as in differentiated melanocytes in the hair follicular bulb (Fig. 2E, lower panels). Similarly to the situation in human melanocytes, immunostaining with the specific anti-Sox9 antibody sc-20095 demonstrated absence of Sox9 expression in X-Gal-positive melanocyte stem cells and their progeny (Fig. 2D, upper and lower panels). Sox9 expression was restricted to cells of the epithelial lineage, namely the outer root sheath and the epithelial stem cell compartment in the bulge area (Fig. 2D; S4 Fig.), in agreement with previous reports [37,38,45].

To address the function of *Sox10* and *Sox9* in the melanocytic lineage *in vivo*, we conditionally ablated either *Sox10* (Fig. 2H) or *Sox9* (Fig. 2F) using *Tyr-CreERT2* transgenic mice [46] carrying floxed alleles of *Sox10* [47] and *Sox9* [27], respectively. Tamoxifen-induced homozygous deletion of *Sox10* in *Sox10^fl/fl^ Tyr-CreERT2* mice resulted in progressive hair graying, revealing an essential function of *Sox10* for melanocyte stem cell homeostasis (Fig. 2I, [23]). In contrast, homozygous deletion of *Sox9* in the melanocytic lineage did not cause hair graying even after a prolonged period after tamoxifen-induced gene deletion (Fig. 2G). Thus, these two closely related genes are not only differentially expressed but also play distinct roles in the biology of melanocytes *in vivo*.

### Sox10 and Sox9 are differentially expressed in mouse giant congenital naevi and melanoma and exhibit functionally distinct roles in tumor initiation

**Figure 3:**
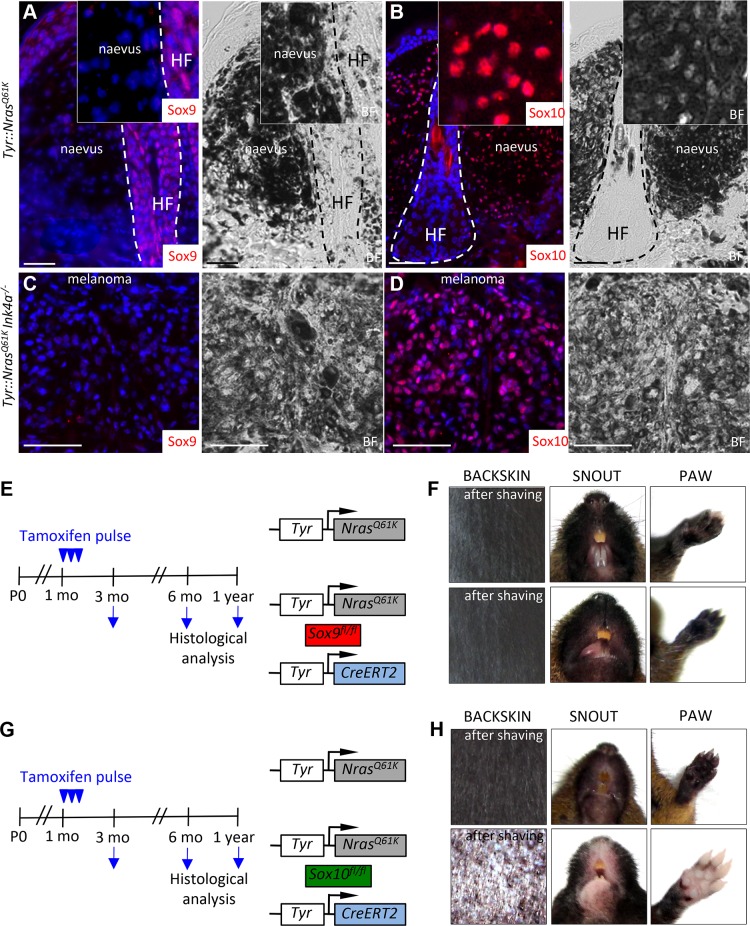
Mouse giant congenital naevi and melanoma reveal no expression of Sox9. **A-D**, Immunostaining for Sox9 (**A, C**) and Sox10 (**B, D**) in the skin sections of *Tyr::Nras^Q61K^* and *Tyr::Nras^Q61K^INK4a^−/−^* mice. **E-H**, Experimental strategy used to abrogate the expression of Sox9 (E) and Sox10 (G) in the mouse melanocytic lineage. Pictures of two representative mice 1 year after tamoxifen injections reveal no reduction in the skin hyperpigmentation in *Tyr::Nras^Q61K^Sox9^fl/+^Tyr-CreERT2* mice as compared to their *Tyr::Nras^Q61K^* littermates (F) in contrast to a pronounced skin whitening observed upon Sox10 loss (H). BF, bright field; HF, hair follicle; mo, months; P0, postnatal day 0. Scale bars, 25 μm.

To functionally assess the role of SoxE factors in melanomagenesis, we first performed immunohistochemical staining for Sox9 and Sox10 of giant congenital naevi formed in *Tyr::Nras^Q61K^* mice and in melanoma derived from giant congenital naevi in *Tyr::Nras^Q61K^Ink4a^−/−^* mice [9,48]. In contrast to the widespread expression of Sox10 displayed by mouse naevi and melanoma tissue (Fig. 3B,D, [9]), immunostaining of both naevi and primary melanoma sections did not reveal any detectable expression of Sox9 protein (Fig. 3A,C; S4B Fig.), consistent with the data that we have obtained for human giant congenital naevi and melanoma (Fig. 1H-I). Despite the lack of detectable Sox9 expression, low levels of Sox9 might be functionally implicated in the formation of melanocytic lesions arising in *Tyr::Nras^Q61K^* mice. To address this issue, we generated *Tyr::Nras^Q61K^ Sox9^fl/fl^ Tyr-CreERT2* mice and conditionally deleted both *Sox9* alleles by tamoxifen treatment of the mice (Fig. 3E). However, skin hyperpigmentation induced by oncogenic NRas was not affected in *Tyr::Nras^Q61K^ Sox9^fl/fl^ Tyr-CreERT2* mice and was comparable to that presented by their control *Tyr::Nras^Q61K^* littermates (Fig. 3F). These data reveal that in contrast to Sox10 (Fig. 3G,H}, Sox9 is not required for the formation of melanocytic lesions.

### Deletion of SOX10 in giant congenital naevus cells results in the induction of SOX9 expression

**Figure 4:**
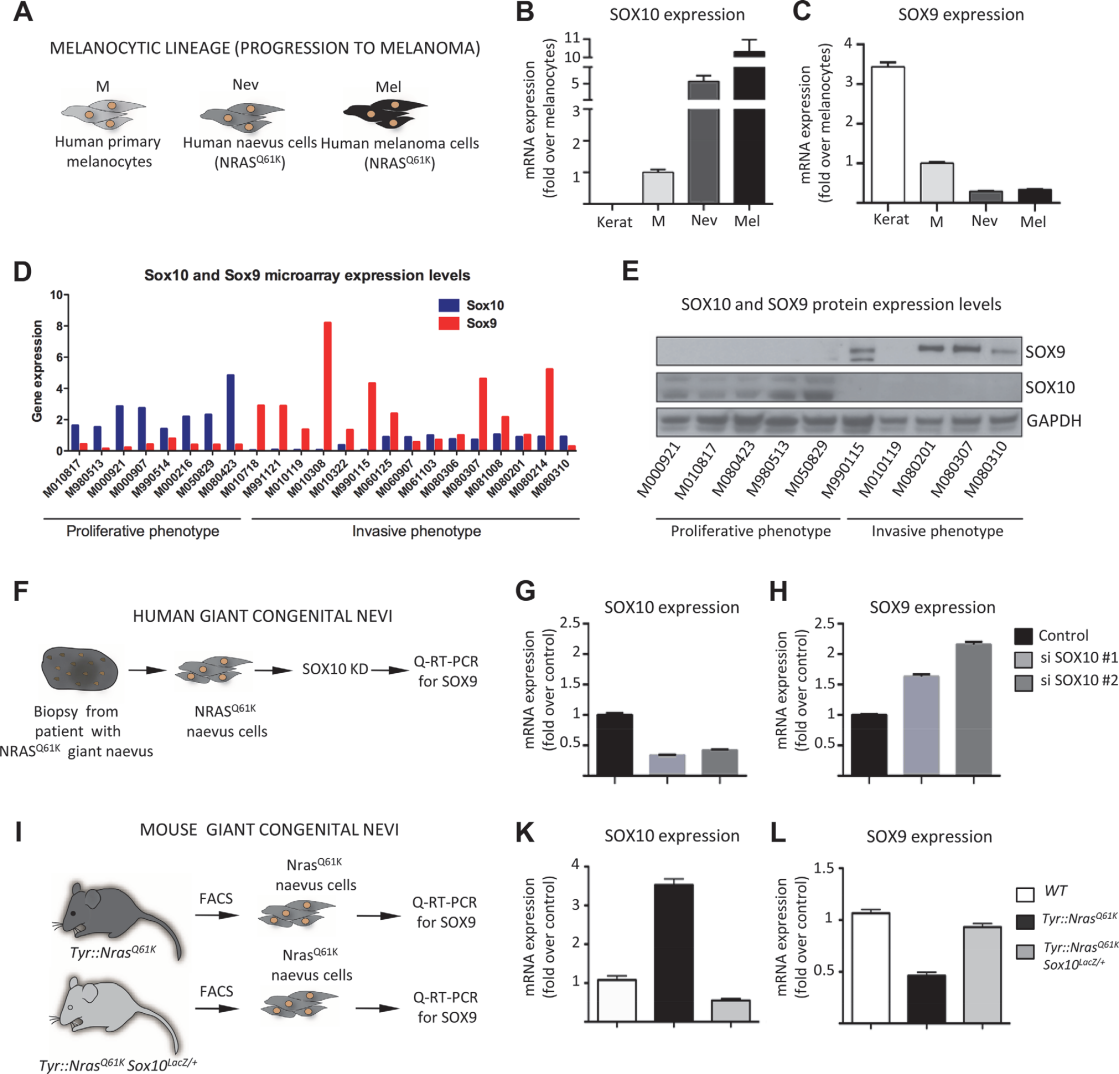
SOX10 knockdown results in elevated SOX9 expression in mouse and human melanocytes. **A**, Experimental design used to investigate the level of SOX9 and SOX10 expression *in vitro*. Cultured human keratinocytes, melanocytes, cells derived from biopsies of patients with giant congential naevi and melanoma cells (M010817 cell line) were subjected to RNA isolation and subsequent Q-RT-PCR analysis. Keratinocytes were used as a control. **B, C**, Quantitative real-time PCR analysis showing the decline of SOX9 expression (**C**) and increase of SOX10 expression (**B**) that correlate with the acquisition of malignant state by human NRAS^Q61K^-mutated cells. Data are presented as the mean fold change and are normalized over levels found in melanocytes. **D, E**, SOX10 and SOX9 expression in a large set of proliferative and invasive cell lines analysed by gene expression using microarrays (**D**) and Western blot (**E**) techniques. **F**, Experimental design used to deregulate SOX10 expression in human cells derived from the biopsy of a patient with NRAS^Q61K^-mutated giant congenital naevus. **G, H**, Quantitative real-time PCR analysis of SOX10 (**G**) and SOX9 (**H**) expression after the knockdown of SOX10. **I**, Experimental design used to analyze the expression of Sox9 in the melanocytic lineage from *Tyr::Nras^Q61K^* and *Tyr::Nras^Q61K^* *Sox10^LacZ/+^* mice. **K, L**, Cells were isolated from the trunk skin of *Tyr::Nras^Q61K^* and *Tyr::Nras^Q61K^* *Sox10^LacZ/+^* mice and stained for Melan-a and c-Kit antibodies. FACS-sorted cells were subsequently used for the RNA isolation and quantitative real-time PCR with primers specific for the coding regions of *Sox9* gene. Data are presented as the mean fold change and are normalized to the control. Kerat, keratinocytes; M, melanocytes; Nev, naevus cells; Mel, melanoma cells; KD, knock down.

To gain insight into the possible interplay between SOX10 and SOX9 during tumor progression, we quantified the expression levels of *SOX10* and *SOX9* in normal human melanocytes, in cells from giant congenital naevi, and in a melanoma cell line (M010817; [49]) using quantitative RT-PCR analysis (Fig. 4A-C). Notably, *NRAS^Q61K^*-associated tumor progression was associated with an increase in *SOX10* expression (Fig. 4B). Cells from giant congenital naevi showed a 5-fold increase in *SOX10* expression when compared to normal melanocytes, while M010817 melanoma cells displayed a 10-fold increase in *SOX10* expression when compared to normal melanocytes (Fig. 4B). In striking contrast to the increase in *SOX10* expression, *SOX9* expression levels were low in human melanocytes and further decreased with melanoma progression (Fig. 4C).

To assess whether the disparate expression of SOX9 and SOX10 is a general feature of human melanoma samples, we analyzed the endogenous expression of these SOXE proteins in a large set of human melanoma cell lines previously categorized into cells with proliferative and invasive signatures, respectively [49]. Of note, all cell lines with a proliferative signature were characterized by high SOX10/low SOX9 mRNA expression (Fig. 4D). However, many cell lines with an invasive signature displayed the opposite expression pattern and showed low SOX10/high SOX9 mRNA expression. These date were confirmed on the protein level by Western blot analysis of several proliferative and invasive cell lines (Fig. 4E).

Interestingly, an inverse correlation between *SOX10* and *SOX9* expression has previously been observed in several systems, including cultured human melanocytes, where upon the induction of differentiation, SOX10 levels were reduced concomitantly with an increase in SOX9 levels [32]. Thus, high expression of a given SoxE transcription factor might be causative for reduced expression of the related SoxE factor. We therefore asked whether deregulation of SOX10 leads to changes in SOX9 expression and found a significant upregulation of SOX9 mRNA expression upon SOX10 knockdown in human giant congenital naevus cells (Fig. 4F-H). This is in analogy to the upregulation of SOX9 mRNA previously observed in melanoma cells upon SOX10 knockdown [9]. Moreover, SOX10 knockdown also resulted in upregulation of SOX9 protein levels in human melanoma cells (S5A Fig.). The combined data indicate that SOX10 normally suppresses SOX9 expression in cells from melanocytic lesions.

To address whether the findings in human cells *in vitro* also apply to the *in vivo* situation in mice, we isolated melanocytic progenitors using fluorescence-activated cell sorting (FACS) from the skin of *Tyr::Nras^Q61K^* and *Tyr::Nras^Q61K^ Sox10^LacZ/+^* mice that lack one allele of *Sox10* (Fig. 4I) and subsequently measured Sox9 expression levels using quantitative RT-PCR (Fig. 4K, L). Elevated Sox10 levels mediated by oncogenic NRas in melanocytic cells from *Tyr::Nras^Q61K^* mice [9] were associated with decreased Sox9 mRNA expression as compared to normal melanoblasts wild-type for NRas. However, reduced Sox10 levels brought about by Sox10 heterozygosity resulted in upregulation of Sox9 expression, as revealed by comparing cells from *Tyr::Nras^Q61K^* with cells from *Tyr::Nras^Q61K^ Sox10^LacZ/+^* mice. Thus, in cells derived from both human and mouse melanocytic lesions, reduced Sox10 levels were accompanied by elevated Sox9 expression.

### SOX10 and SOX9 display antagonistic functions in melanoma cells

**Figure 5:**
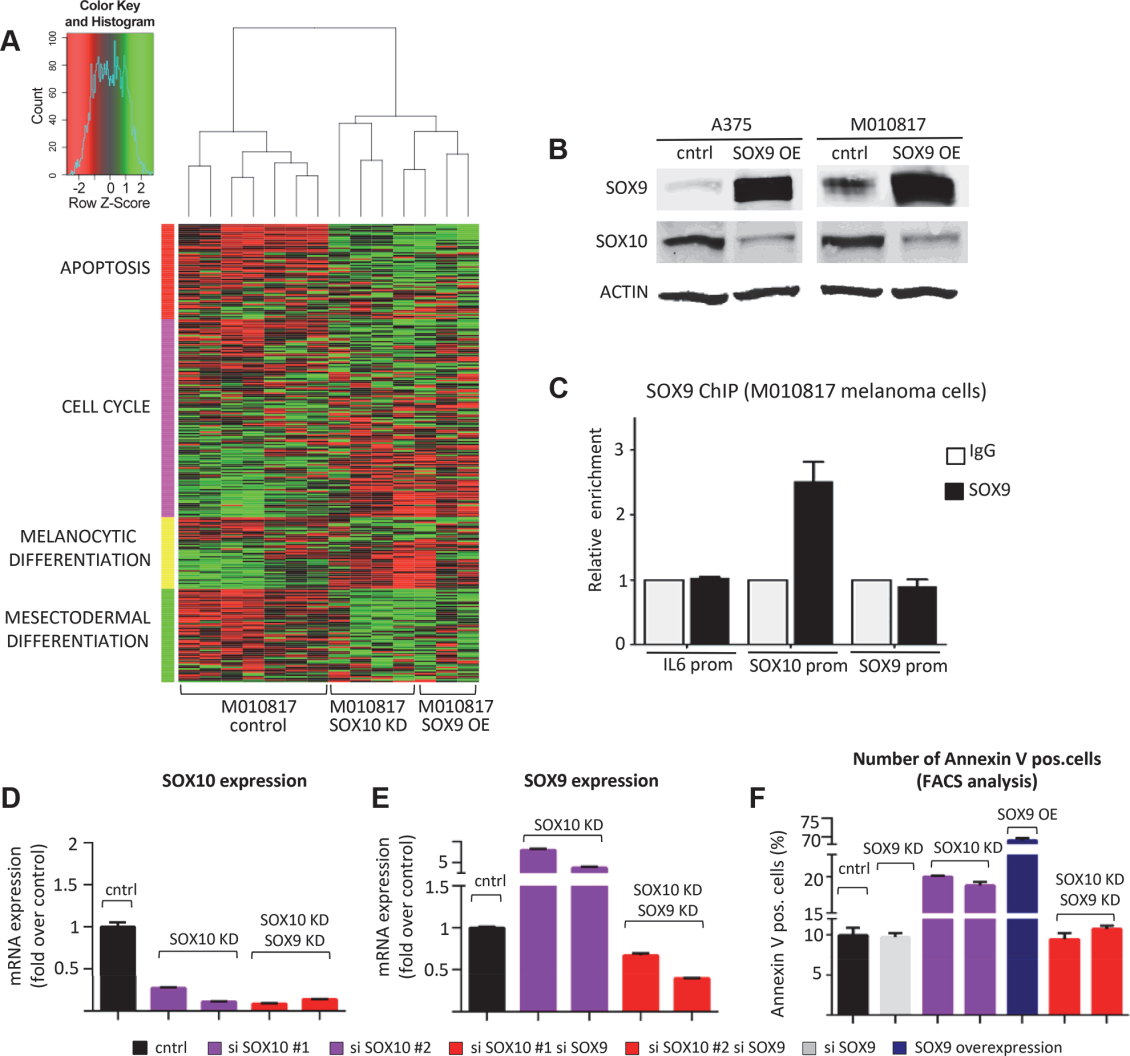
Experimental suppression of SOX9 expression rescues the effects of SOX10 deregulation in human melanoma cells. **A**, SOX9 overexpression in human melanoma cells closely resembles the gene expression signature of SOX10 knockdown as revealed by unsupervised hierarchical clustering of control M010817 melanoma cells, SOX9 overexpressing M010817 cells and SOX10 knock down M010817 cells. Microarray gene expression accession number: GSE37059. **B**, Western blot analysis showing that SOX10 expression is downregulated upon overexpression of SOX9 in two independent human melanoma cell lines (A375 and M010817). **C**, Chromatin immunoprecipitation assay demonstrating the binding of SOX9 to the promoter of SOX10 in human melanoma M010817 cells. **D, E**, Quantitative real-time PCR analysis of SOX10 (**E**) and SOX9 (**F**) expression after the knockdown of SOX10 and after the double knockdown of SOX10 and SOX9 in M010817 cell line. **F**, Quantification of number of Annexin V-positive cells based on the FACS analysis in the melanoma M010817 cells upon SOX9 KD, SOX10 KD or double SOX9/SOX10 KD. OE, overexpression; KD, knock down; ChIP, chromatin immunoprecipitation; prom, promoter.

Based on our findings it is conceivable that increased levels of SOX9 might mediate at least some of the anti-tumorigenic effects observed upon SOX10 loss-of-function in melanoma. To address this hypothesis, we overexpressed SOX9 in human M010817 melanoma cells *in vitro* and compared the gene expression profile of these cells with that of *SOX10* knock-down melanoma cells, using the parental M010817 cell line as control [9]. Unsupervised hierarchical clustering revealed that overexpression of *SOX9* led to a gene expression profile closely resembling the *SOX10* knockdown-signature, which included in both conditions regulators of cell cycle progression, apoptosis, and melanocytic and mesectodermal differentiation (Fig. 5A). Among others, these data suggest that while SOX10 acts as an inhibitor of apoptosis in melanoma cells, SOX9 elicits a proapoptotic response. Similarly, SOX10 and SOX9 appear to play antagonistic functions in the regulation of the cell cycle, melanocytic differentiation, and mesectodermal differentiation (Fig. 5A).

Notably, *SOX9* overexpression resulted in decreased expression of a number of genes associated with melanocytic differentiation, such as MLANA, MITF, DCT, TYR, and importantly SOX10. To confirm the downregulation of SOX10 upon SOX9 overexpression observed in microarray analysis (Fig. 5A) also on the protein level, we performed Western blot analysis and observed a pronounced downregulation of SOX10 protein upon SOX9 overexpression in human melanoma cell lines (Fig. 5B). Moreover, chromatin immunoprecipitation assays in human melanoma M010817 cells indicated that SOX9 binds to the promoter of SOX10, suggesting a direct regulation of the *SOX10* gene by SOX9 (Fig. 5C). Thus, whereas SOX10 loss-of-function leads to increased SOX9 expression (Fig. 4), high levels of SOX9 suppress SOX10 expression, revealing cross-regulatory interactions between these two transcription factors.

Next, we addressed whether the cross-regulation between SOX10 and SOX9 is functionally relevant in human melanoma cells. To this end, we performed RNAi experiments to test whether interfering with SOX9 overexpression upon SOX10 knockdown could rescue M010817 melanoma cells (Fig. 5D-F). Using two independent sets of siRNAs, the elevated SOX9 levels observed in SOX10 knockdown cells were reverted below control levels by means of concomitant SOX9 knockdown (Fig. 5E). Importantly, both SOX9 gain-of-function and SOX10 knockdown promoted apoptosis (Fig. 5F; Fig. S5B). However, SOX10 knockdown cells were rescued when SOX9 expression was simultaneously downregulated, resulting in numbers of apoptotic cells comparable to those found in control cells (number of Annexin V-positive apoptotic M010817 cells: control, 9.95±0.9%; SOX10 siRNA #1, 19.96±0.13%; SOX10 siRNA #2 18.8±0.49%; combination of SOX10 siRNA #1/SOX9 siRNA, 9.45±0.79%; combination of SOX10 siRNA #2/SOX9 siRNA. 10.75±0.4%) (Fig. 5F; S5B Fig.). These data indicate that at least *in vitro*, SOX9 plays a key role in mediating the cellular phenotype obtained in human melanoma cells upon suppression of SOX10.

### Deletion of Sox9 rescues the phenotype of Tyr::Nras^Q61K^Sox10^fl/+^Tyr-CreERT2 mice, restoring Nras^Q61K^-mediated naevus formation

**Figure 6:**
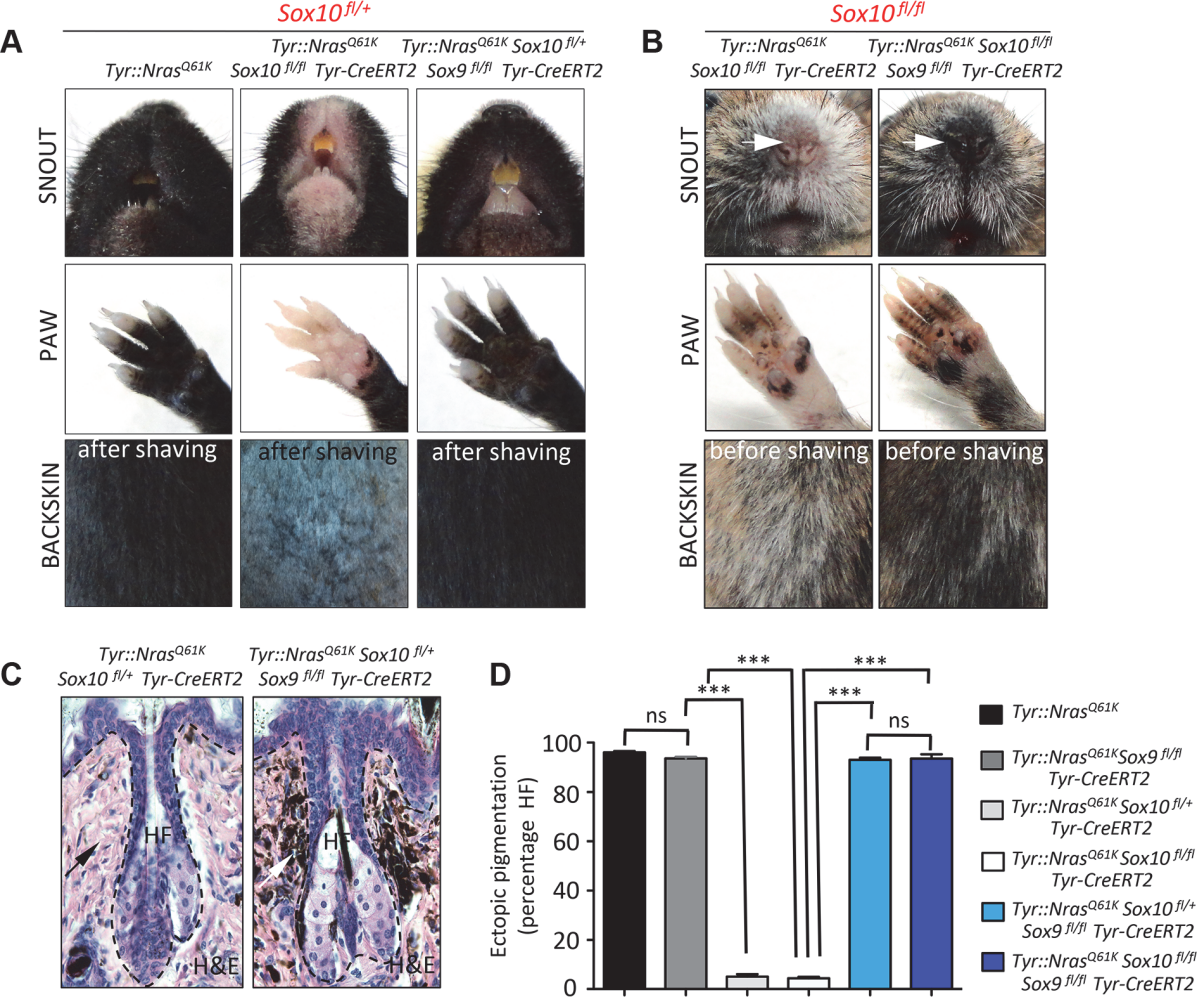
Homozygous deletion of *Sox9* rescues the effects of Sox10 loss in *Tyr::Nras^Q61K^* mice and restores hyperpigmentation *in vivo*. **A, B**, Representative pictures of back skin, paws and snouts from mice of the indicated genotypes. **C, D**, Histological evaluation of the hyperpigmentation phenotype in the skin. Haematoxylin and eosin staining of back skin (**C**) was followed by the quantification (**D**) of the percentage of hair follicles associated with the hyperpigmentation. HF, hair follicle; H&E, haematoxylin and eosin.

As in human melanoma cells *in vitro*, reducing Sox10 levels *in vivo* elicits an anti-tumorigenic effect, preventing melanocytic hyperplasia in *Tyr::Nras^Q61K^Sox10^fl/+^Tyr-CreERT2* mice [9]. To functionally test whether upregulation of Sox9 is the key factor accountable for the lack of hyperplasia in these mice *in vivo*, we performed simultaneous conditional ablation of both *Sox10* and *Sox9* genes in the *Tyr::Nras^Q61K^* mice (Fig. 6A, B). In agreement with our previous observations [9], the skin of the snout, paws and the back was noticeably lighter in color in *Tyr::Nras^Q61K^Sox10^fl/+^Tyr-CreERT2* mice as compared to their control *Tyr::Nras^Q61K^* littermates (Fig. 6A, left and middle panels). In contrast, macroscopic examination of the skin of *Tyr::Nras^Q61K^Sox10^fl/+^Sox9^fl/fl^Tyr-CreERT2* animals showed pronounced hyperpigmentation, a hallmark of the skin phenotype found in *Tyr::Nras^Q61K^* mice (Fig. 6A, right panels). Thus, conditional deletion of *Sox9* rescued the effect of *Sox10* haploinsufficiency in *Tyr::Nras^Q61K^* mice.

As shown in Fig. 2, conditional ablation of both alleles of *Sox10* in the normal melanocytic lineage using *Tyr-CreERT2* resulted in loss of functional melanocytes and hair graying (Fig. 2I). Likewise, homozygous deletion of *Sox10* in *Tyr::Nras^Q61K^* mice not only prevented *Nras^Q61K^*-stimulated skin hyperpigmentation, but also led to hair greying in these mice (Fig. 6B, left panels). To determine whether these phenotypes involve Sox9 activity and whether, therefore, loss of *Sox9* could rescue the effects of homozygous *Sox10* deletion, we generated *Tyr::Nras^Q61K^Sox10^fl/fl^Sox9^fl/fl^Tyr-CreERT2* animals. Surprisingly, loss of *Sox9* in *Sox10* homozygous conditional knock-out animals efficiently restored skin hyperpigmentation as seen on the snout and paws of *Tyr::Nras^Q61K^Sox10^fl/fl^Sox9^fl/fl^Tyr-CreERT2* animals when compared to mice lacking *Sox10* but not *Sox9* in the melanocytic lineage (Fig. 6B, right panels).

We have previously demonstrated that skin hyperpigmentation in *Tyr::Nras^Q61K^* mice is due to hyperplasia of ectopically located pigment cells emerging in the dermis, around the upper part of the hair follicle [9]. To measure the degree of hyperpigmentation in *Tyr::Nras^Q61K^* mice with conditional *Sox10* deletion (heterozygous and homozygous) versus mice simultaneously lacking both *Sox9* alleles and one or both *Sox10* alleles in the melanocytic lineage, we quantified the percentage of hair follicles associated with ectopically located melanocytic cells in the back skin of these mouse lines. In *Tyr::Nras^Q61K^* mice, more than 90% of all hair follicles displayed ectopic pigment cells (Fig. 6D). In contrast, in the absence of one or both alleles of *Sox10*, there were almost no hair follicles with ectopic pigment cells, despite Nras^Q61K^ expression (Fig. 6C, D). Strikingly, however, the percentage of hair follicles associated with ectopic melanoblasts in *Tyr::Nras^Q61K^Sox10^fl/+^Sox9^fl/fl^Tyr-CreERT2* animals was reverted to numbers similar to those found in *Tyr::Nras^Q61K^* mice (93±1.8% and 96±1%, respectively) (Fig. 6C, D). Moreover, even in the absence of both *Sox10* alleles, loss of *Sox9* rescued the Nras^Q61K^–dependent appearance of melanocytic cells found outside hair follicles (93.5±3%) (Fig. 6D). These data reveal a key role of Sox9 in preventing melanoma initiation and provide novel insights into the functional interplay between Sox10 and Sox9 during melanoma formation.

## Discussion

Our study identifies the structurally related transcription factors SOX10 and SOX9 as functionally antagonistic regulators of postnatal melanocyte and melanoma development. Although we did not find SOX9 to be expressed in the melanocytic lineage when SOX10 is present, SOX9 expression becomes evident upon SOX10 inactivation in naevus and melanoma cells. In this context, SOX9 appears to promote the major cellular processes induced by SOX10 loss-of-function, namely stop of proliferation and apoptosis. Intriguingly, SOX9 and SOX10 are engaged in a cross-regulatory feedback loop whereby SOX9, which is induced upon SOX10 inactivation, itself suppresses SOX10, thus strengthening an anti-tumorigenic program.

In many cell lineages and tissues, SOX10 and SOX9 are co-expressed and functionally redundant [50]. We propose that this is not the case in melanocytic cells and that SOX9, unlike SOX10, is neither required for normal melanocyte stem cell homeostasis nor for formation of congenital nevi and primary melanoma. Our findings disagree with some previously published studies reporting SOX9 expression in the normal and tumor-associated melanocytic lineage [32–34,36,51]. However, as we demonstrate here, most previously used anti-SOX9 antibodies display cross-reactivity with SOX10, owing to the close relationship between these two SoxE factors. Having identified anti-SOX10 and anti-SOX9 specific antibodies, we reveal virtually exclusive expression patterns of these transcription factors in the normal human skin and in a large set of melanoma biopsies and cell lines. While SOX10 expression is restricted to neural crest derivatives, including melanoblasts, differentiated melanocytes, and virtually all human naevus and melanoma biopsies tested (Fig. 1; S3 Fig.; [9], SOX9 expression in melanocytic cells was restricted to few scattered cells in a subset of melanoma biopsies. In contrast, SOX9 was strongly expressed in epithelial cells of the hair follicle, which are devoid of SOX10 expression.

In support of these data, Sox10 protein expression in the mouse skin is detected *in vivo* throughout all stages of melanocyte development from stem cells to differentiated melanocytes in the hair follicular bulb (Fig. 2). Mice lacking *Sox10* in the melanocyte lineage display hair graying, indicating that Sox10 is necessary for maintenance of melanocyte stem cells and committed melanoblasts [23] (Fig. 2). Likewise, Sox10 is required for the establishment of giant congenital naevi as well as melanoma [9]. In contrast, murine Sox9 appeared not to be expressed in melanocytic cells of the normal skin, nevi, and primary melanoma, while it was readily detectable in epithelial cells in accordance with previous reports [33,37,38]. Importantly, loss of function analyses failed to reveal a crucial role of *Sox9* in normal melanocytes, as conditional deletion of *Sox9* did not affect generation and long-term maintenance of melanocytes *in vivo* and did not result in hair graying, a phenotype characteristic for the loss of *Sox10*. Likewise, lack of *Sox9* did not prevent emergence of melanocytic lesions induced by oncogenic NRas^Q61K^ (Fig. 3). These data demonstrate that Sox10 and Sox9 are not only expressed in different cellular compartments in the skin, but also play distinct roles in normal and transformed melanocytes.

In cell types other than melanocytes Sox9 and Sox10 can act redundantly. For instance, in oligodendroglial progenitors, concomitant expression of Sox9 can compensate for the loss of Sox10 [52,53]. Similarly, in avian and *Xenopus* embryos, Sox9 and Sox10 are co-expressed in premigratory neural crest cells and are both able to induce ectopic neural crest cell formation upon forced expression in chicken neural tube [11,24,54]. In addition, the two factors are able to cross-regulate each other at this early stage of neural crest formation. Interfering with Sox10 function leads to inhibition of Sox9 expression [55], suggesting that Sox10 is required for the expression of Sox9 in pre-migratory neural crest. On the other hand, Sox9 overexpression in *Xenopus* embryos leads to upregulation of Sox10 expression [24], suggesting that Sox9 can also act upstream of Sox10. As development proceeds, however, Sox10 expression persists in the trunk neural crest and is downregulated in cranial neural crest cells giving rise to mesectodermal structures, while Sox9 expression is absent in trunk neural crest cells but present in the cranial neural crest [24,26]. These divergent expression patterns are established by signaling pathways differentially regulating transcription of Sox9 and Sox10, respectively. In particular, TGFβ (transforming growth factor β) simultaneously triggers induction of Sox9 and reduction of Sox10 expression [56]. Accordingly, mice lacking *Sox10* display phenotypes that are distinct from those obtained upon loss of *Sox9* [13,54,56–58]. In particular, Sox10 but not Sox9 is expressed in and required for the generation of melanoblasts during mouse embryogenesis [13, 21]. Finally, in agreement with our expression studies on human skin, humans carrying mutations in *SOX9* display campomelic dysplasia affecting the skeleton and reproductive system but not melanocytes, whereas patients with mutations in *SOX10* often exhibit pigmentary anomalies [20,50,59].

However, the divergent functions of Sox10 and Sox9 in the skin appear not to be simply due to their differential expression patterns. Depending on the cellular context, these two transcription factors can also elicit different responses in one and the same cell lineage rather than playing redundant roles. Studies in *Xenopus* embryos demonstrated that while expression of Sox10 at the two-cell stage was sufficient to activate the expression of Trp-2 (Dct) and the induction of melanocytic precursors, the expression of Sox9 failed to do so [24]. In mice, loss of Sox9 promotes apoptosis and other phenotypes in neural crest cells, but Sox10 is maintained in these cells and cannot rescue the *Sox9*-mutant phenotype [54]. Moreover, while Sox9 activates the expression of genes involved in the induction of osteochondrogenesis in neural crest cells in pharyngeal arches [26,28,60], Sox10 is involved in the specification of a glial and melanocytic gene expression program [13]. In this context, Sox10 and Sox9 play antagonistic roles, in that Sox9 promotes cells cycle exit and mesenchymal fates, while Sox10 activates proliferation and suppresses mesenchymal fate acquisition [56]. Accordingly, *Sox10* inactivation results in induction of Sox9-dependent fates in postmigratory neural crest cells. This interplay between Sox10 and Sox9 functions during normal neural crest development is highly reminiscent of our findings in melanocytic lesions, where Sox10 also promotes proliferation and survival, while Sox9 counteracts these cellular processes. Indeed, in human melanoma cells, loss of SOX10 not only resulted in upregulation of SOX9 expression, but also in global transcriptional changes highly similar to the changes observed upon SOX9 overexpression (Fig. 5), indicating that these factors appear to play opposing functions in melanoma. Interestingly, a study by Passeron et al. revealed that overexpression of SOX9 prevents melanoma formation [35] by increasing the expression of the CDK inhibitor p21 and subsequent cell cycle arrest. Likewise, a recent report by Pavan and colleagues established that the expression of p21 and p27 were increased upon SOX10 knockdown [31]. Thus, our data might provide one explanation for the anti-tumorigenic effect of SOX9, namely by downregulation of SOX10. This is in accordance with our previously published results on the essential role of SOX10 for melanoma initiation and progression [9]. Of note, the anti-tumorigenic effect elicited by suppressing SOX10 was abolished by concomitant SOX9 inactivation both in human melanoma cells as well as in mice. Thus, antagonistic SOX10/SOX9 constitutes a key node in the genetic network underlying melanomagenesis. Nonetheless, it is conceivable that further cues mediate SOX10-pro- and SOX9-anti-tumorigenic effects, respectively. Moreover, our data do not exclude a role of SOX9 at later stages of melanoma disease progression, in particular during metastasis formation by invasive cells. Indeed, while we could attribute a SOX10 high/SOX9 low signature to proliferative human melanoma cell lines and to all human and murine melanoma tissues analyzed, several human melanoma cell lines reported to display invasive features [49] exhibited SOX10 low/SOX9 high expression. Although this remains to be shown, these invasive cell lines with SOX10 low/SOX9 high expression might have been established by capturing or inducing invasive tumor cells that appear to be rather rare in biopsies of bulk tumor tissue. Likewise, apart from experimentally reducing SOX10 levels, other stimuli such as UV exposure might also lead to upregulation of SOX9 [51]. In any case, our discovery of the antagonistic interaction between SOX10 and SOX9, together with the further characterization of their mode of action in melanoma cells, might not only provide new mechanistic insights into how SoxE group proteins are regulated and act in the context of melanoma initiation and maintenance, but might also point to novel strategies for melanoma therapies.

## Materials and Methods

### Human specimens

All analyses involving human skin, giant congenital naevi and melanoma tissue were performed in accordance with the ethical committee in canton Zurich, Switzerland. TMA containing melanoma tissue was constructed as previously described [61].

### Mice

*Tyr::Nras^Q61K^* [48] were provided by F. Beermann (EPFL Lausanne, Switzerland). *Dct-LacZ* mice were described previously [44]. *Sox10^fl/fl^* mice were described previously [47]. *Sox9^fl/fl^* mice [27] were a kind gift from G. Scherer (Institute of Human Genetics, Freiburg, Germany). *Tyr-CreERT2* line [46] was provided by L. Chin (The University of Texas MD Anderson Cancer Center, Houston, Texas, USA). *Rosa26-lacZ* mice were obtained from Jackson laboratory. All animal experiments were performed in accordance with Swiss law and have been approved by the veterinary authorities of Zurich.

### Tamoxifen injections

Mice were subjected to intraperitoneal injections of tamoxifen (T5648, Sigma), diluted with the mixture of ethanol and sunflower oil (1:9 ratio). Tamoxifen was injected for 5 consecutive days.

### Histological analysis and immunohistochemistry

Immunohistochemistry on paraffin sections was performed as previously described [9]. Briefly, skin samples were fixed in 4% buffered paraformaldehyde and embedded in paraffin. For immunohistochemistry, antigen retrieval was performed in citrate buffer (pH 6.0) for 10 minutes at 110°C in HistoPro (Rapid Microwave Histoprocessor, Milestone, USA). The following primary antibodies were used: anti-Sox10 (goat, 1:200, Santa Cruz Biotechnology, Santa Cruz, CA), anti-Sox10 (mouse, 1:200, R&D), anti-Sox9 (rabbit, 1:100, sc-20095, Santa Cruz Biotechnology, Santa Cruz, CA), anti-Sox9 (rabbit, 1:100, ab36748, Abcam), anti-Sox9 (M00006662, Abnova), anti-Sox9 (AB5535, Millipore), anti-Sox9 (GTX 109661, GenTex), anti-MITF (mouse, clone 6D3, 1:500) was a kind gift from Heinz Arnheiter (NIH, USA). Images were captured with a Leica DMI 6000B Microscope and using LAS AF (Leica Application Suite Advanced Fluorescence) software. For whole mount X-Gal staining, skin samples were fixed with 4% buffered paraformaldehyde, washed with PBS and subjected to X-Gal staining solution overnight at 37°C. After several washing steps, tissue was paraffin embedded and sectioned. 5 μm thick sections were further counterstained with eosin solution and mounted.

### RNA isolation, reverse transcription and quantitative PCR

Total RNA was isolated using Trizol according to manufacturer’s instructions (Invitrogen). 1 μg aliquots of RNA were reverse transcribed with Reverse Transcription System (Promega) according to the manufacturer’s instructions. Data collection and analysis were performed by ABI Viia7 Fast Real-Time PCR Systems (Applied Biosystems). Gene expression values of averaged triplicate reactions were normalized to *RPL28* expression levels. RPL28 primers are as follows: 5’-GCAATTGGTTCCGCTACAAC-3’ and 5’-TGTTCTTGCGGATCATGTGT-3’. The expression of SOX10 and SOX9 was measured using primers purchased from QIAGEN: SOX10 (Hs_SOX10_1_SG); SOX9 (Hs_SOX9_1_SG).

### Sequencing

Cells derived from patients with giant congenital naevi were sequenced for NRAS. Primers for sequencing for Exon 1 (mutation G12) and Exon 2 (mutation Q61K) of NRAS gene were as follows: NRAS_1F 5’-ATAGAAAGCTTTAAAGTACTG-3’ and NRAS_1R 5’-TTCCTTTAATACAGAATATGG-3’, NRAS_2F 5’-CCCCTTACCCTCCACAC-3’ and NRAS_2R 5’-AACCTAAAACCAACTCTTCCCA-3’.

### Cell culture and transfection assays

Silencing RNA (siRNA) transfection was carried out using INTERFERin transfection solution according to the manufacturer’s protocol (Polyplus-transfection, Illkirch, France). Cells were transfected with 10 nM of siRNA (Qiagen) for 96 hours before RNA was extracted or used for FACS analysis. As control siRNA, the All-Star negative siRNA sequence (Qiagen) was used, and gene-specific siRNAs targeting siSOX10 (SI00729414, SI00729421) and siSOX9 (SI00007595, SI00007609) were obtained from Qiagen. Transfection of DNA was carried using JetPEI transfection solution according to the manufacturer’s protocol (Polyplus-transfection, Illkirch, France). Cells were transfected with 1 ug of pCMV6-SOX9 (Origene SC321884) or empty vector for 96 hours before RNA was extracted or used for FACS analysis.

### Melanocyte FACS and RNA seq

Melanocytes were purified by FACS from doxycycline-treated iDct-GFP mice as previously described [41]. Total RNA was prepared from FACS-sorted cell fractions containing GFP-positive melanoblasts/melanocytes according to standard Illumina RNA-Seq paired-end protocol and sequenced on the Illumina GAIIx to 80 bp per read.

### Microarray analysis

Total RNA was isolated from melanoma cell cultures using TRIzol according to the manufacturer’s instructions (Invitrogen). Total RNA was amplified and biotin-labelled using the Message Amp II-Biotin Enhanced aRNA Amplification Kit (Ambion, Austin, TX, USA). Biotin-labelled RNA was hybridized to Affymetrix HG-U133 plus 2.0 oligonucleotide microarrays following the manufacturer’s protocol (Affymetrix, Santa Clara, CA, USA). After hybridization, microarrays were washed and stained using a GeneChip Fluidics Station 450 (Affymetrix) and then scanned using a GeneChip Scanner 7G (Affymetrix). Raw data was processed by R using the affycoretools package (RMA). Gene expression datasets for SOX10 knockdown were obtained from NCBI GEO GSE37059. Gene expression analysis was performed by R using the limma package. P-values were adjusted by FDR p-value adjustment. For melanoma cell lines analysis (proliferative vs invasive): Normalized expression values were downloaded from GSE4840 containing microarray data for twenty three melanoma cell cultures. Pearson’s product moment correlation (r) was calculated for the SOX10 and SOX9 expression values across all twenty three samples. P-value was determined from the t statistic calculated from r.

### Flow cytometry and cell sorting

Skin tissue (from back skin) was digested with a mixture of Dispase (Roche) and Collagenase I (Worthington) for 1 hour at 37°C and enzymatic reaction was stopped by addition of DMEM media supplemented with 10%FCS as previously described [9]. Subsequently, single cell suspension was filtered through 40 μm strainers (BD). For cell cycle analysis, Click-iT EdU Alexa Fluor 647 Flow Cytometry Assay Kit (Invitrogen) was used. Cells were labeled with PI according to manufacturer’s protocol and DNA content was measured using a BD FACSCanto II flow cytometer (BD Biosciences) and a BD FACSDiva software (BD Biosciences). For measurement of apoptosis, Annexin V-PE Apoptosis Detection Kit I (BD Pharmingen, 559763) was used. FACSAria sorter and FACS DiVa software (BD Biosciences) were used for cell sorting.

### Chromatin immunoprecipitation

ChIP analysis was performed as previously described [62]. Sox9 antibody was from Santa Cruz Biotechnology (sc-20095, Santa Cruz Biotechnology). SOX10 promoter sequences were amplified with forward primer (5’-CCTCTGCCTCGTGTGACTAC-3’) and reverse primer (5’-TCCTGTCTGGAGTGGGCTG-3’).

## Supporting Information

S1 Fig Immunohistochemical analysis of the cross-reactivity of anti-Sox9 antibodies to Sox10 in the mouse skin. **A-D**, Immunostaining for Sox9 (red) using different anti-Sox9 antibodies, namely anti-Sox9 from Santa Cruz (A), Millipore (B), Abcam (C) and Abnova (D). **E**, Summary of immunohistochemical analysis using anti-Sox9 antibodies from different sources. ORS, outer rooth sheath; M, melanocytes.
(PPSX)

S2 Fig Assessing the cross-reactivity of anti-SOX9 antibodies to SOX10. **A-D**, Western blots demonstrating the cross-reactivity of anti-SOX9 antibodies (Abcam, Abnova and Millipore) to SOX10 and specificity to SOX9 (A-D). **E**, A schematic illustration of the experiment used to test anti-SOX9 antibodies. F-K, Western blots demonstrating the cross-reactivity of anti-SOX9 antibodies using protein homogenates isolated from melanoma cells transfected with either sh control or sh SOX10 vectors.
(PPTX)

S3 Fig SOX10 and SOX9 expression in human melanocytic and epithelial lineages. **A**, A schematic representation of the location of melanocytes in the hair follicular bulb. **B**, SOX10 expression in the hair follicular bulb. **C**, SOX9 expression in human basal cell carcinoma. **D**, Analysis of the expression of SOX10 and SOX9 in the human giant congenital naevi (patient H08 10533). Adjacent sections were stained with anti-SOX10 and anti-SOX9 antibodies. Note the positive staining for SOX9 in the hair follicle. BCC, basal cell carcinoma; GCMN, giant congenital melanocytic naevi; M, melanocytes.
(PPTX)

S4 Fig SOX9 is not expressed in the murine melanocytes and cells of giant congenital naevi in the postnatal mouse skin. **A**, Bright field picture (left panel) showing the pigmented melanocytes located in the hair follicular bulb. Immunostaining for SOX9 (red) demonstrating that SOX9 is expressed in the epithelial cells of the hair follicle (outer root sheath) but not in the pigmented melanocytes. **B**, Immunostaining for Sox9 (red) demonstrating the expression of Sox9 in the outer rooth sheath and the absence of Sox9 expression in the cells of giant congenital naevi in *Tyr::Nras^Q61K^* mouse. BF, bright field; HF, hair follicle, M, melanocytes; ORS, outer root sheath.
(PPTX)

S5 Fig SOX9 and SOX10 play antagonistic roles in human melanoma cells. **A**, Western blot analysis demonstrating that SOX9 expression is upregulated upon SOX10 knockdown in human melanoma cell lines. **B**, FACS analysis of apoptosis in M010817 melanoma cell line. M010817 control cells, M010817 SOX10 KD cells, M010817 SOX9 OE and M010817 SOX10 KD SOX9KD cells were analyzed for the number of Annexin V-positive cells. KD, knockdown; OE, overexpression.
(PPTX)
