# Supplementary figures and images for: Antagonistic Cross-Regulation between Sox9 and Sox10 Controls an Anti-tumorigenic Program in Melanoma
Source: PLoS Genet. 2015 Jan 28;11(1):e1004877. doi: 10.1371/journal.pgen.1004877 (PMC4309598; doi:10.1371/journal.pgen.1004877)

## Slide 1
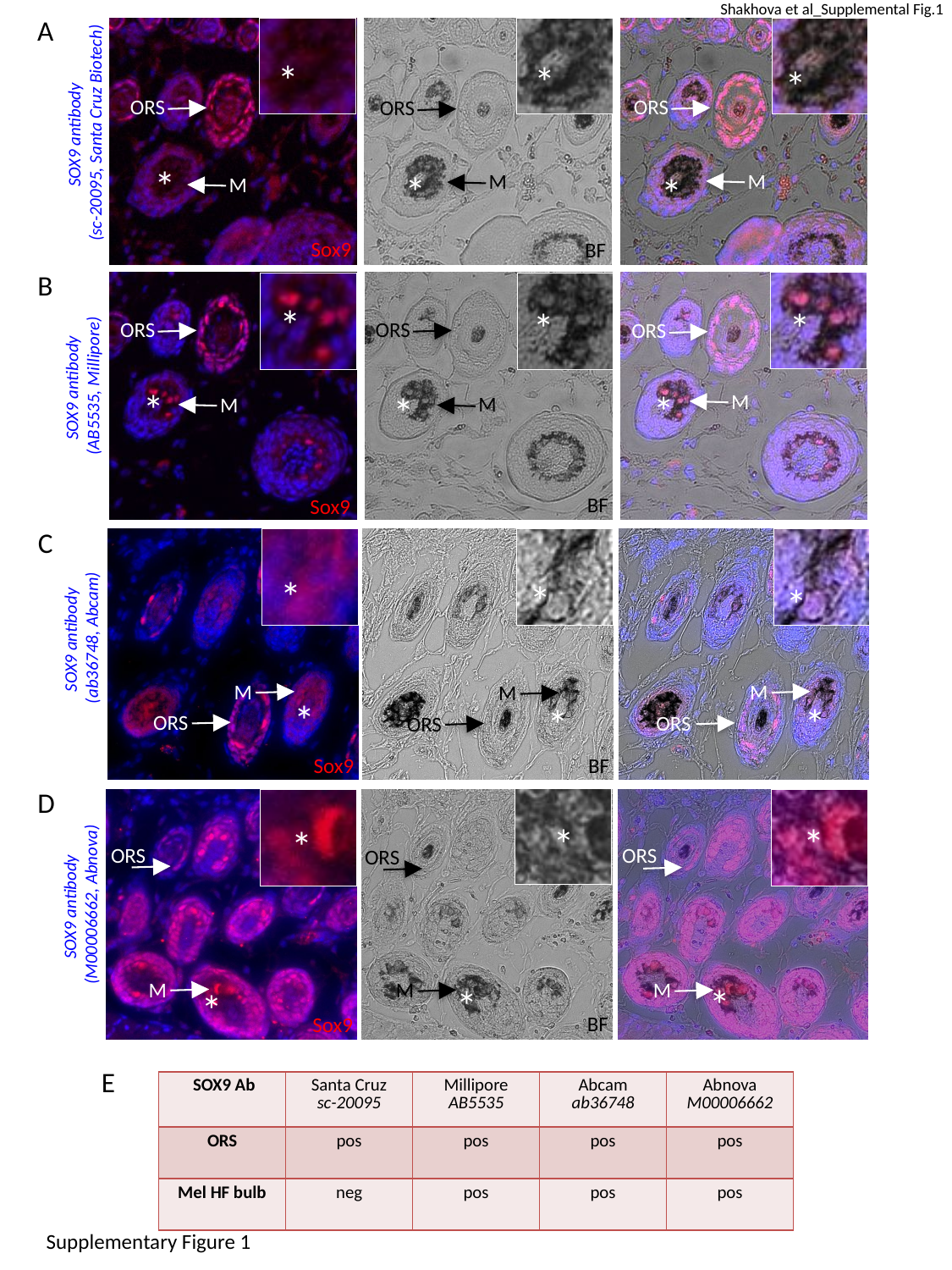

Supplement: S1 Fig — A-D, Immunostaining for Sox9 (red) using different anti-Sox9 antibodies, namely anti-Sox9 from Santa Cruz (A), Millipore (B), Abcam (C) and Abnova (D). E, Summary of immunohistochemical analysis using anti-Sox9 antibodies from different sources. ORS, outer rooth sheath; M, melanocytes. (PPSX) [file pgen.1004877.s001.ppsx]
