# Supplementary material for: Antagonistic Cross-Regulation between Sox9 and Sox10 Controls an Anti-tumorigenic Program in Melanoma
Source: PLoS Genet. 2015 Jan 28;11(1):e1004877. doi: 10.1371/journal.pgen.1004877 (PMC4309598; doi:10.1371/journal.pgen.1004877)
Supplement: S2 Fig — A-D, Western blots demonstrating the cross-reactivity of anti-SOX9 antibodies (Abcam, Abnova and Millipore) to SOX10 and specificity to SOX9 (A-D). E, A schematic illustration of the experiment used to test anti-SOX9 antibodies. F-K, Western blots demonstrating the cross-reactivity of anti-SOX9 antibodies using protein homogenates isolated from melanoma cells transfected with either sh control or sh SOX10 vectors. (PPTX) [file pgen.1004877.s002.pptx]

## Slide 1
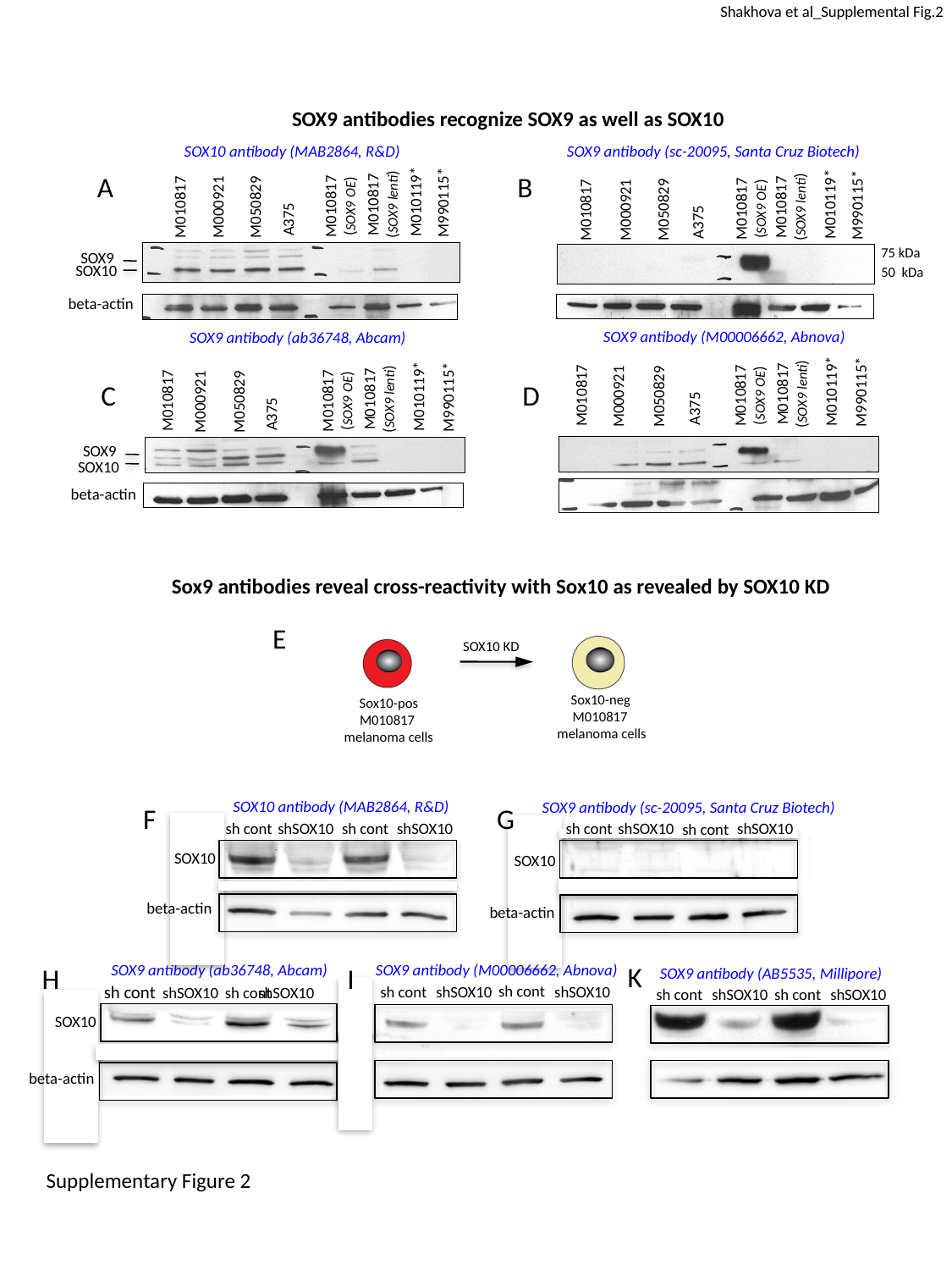

Shakhova et al_Supplemental Fig.2
SOX9 antibodies recognize SOX9 as well as SOX10
SOX10 antibody (MAB2864, R&D)
SOX9 antibody (sc-20095, Santa Cruz Biotech)
A
B
M010817
(SOX9 lenti)
M010817
(SOX9 OE)
M010817
(SOX9 lenti)
M010817
(SOX9 OE)
M010119*
M990115*
M010119*
M990115*
M010817
M000921
M050829
M010817
M000921
M050829
A375
A375
75 kDa
SOX9
SOX10
50 kDa
beta-actin
SOX9 antibody (M00006662, Abnova)
SOX9 antibody (ab36748, Abcam)
M010817
(SOX9 lenti)
M010817
(SOX9 OE)
C
D
M010817
(SOX9 lenti)
M010119*
M010817
(SOX9 OE)
M990115*
M010817
M010119*
M000921
M050829
M990115*
M010817
M000921
M050829
A375
A375
SOX9
SOX10
beta-actin
Sox9 antibodies reveal cross-reactivity with Sox10 as revealed by SOX10 KD
E
SOX10 KD
Sox10-neg
M010817
melanoma cells
Sox10-pos
M010817
melanoma cells
SOX10 antibody (MAB2864, R&D)
SOX9 antibody (sc-20095, Santa Cruz Biotech)
F
G
shSOX10
sh cont
shSOX10
sh cont
shSOX10
sh cont
shSOX10
sh cont
SOX10
SOX10
beta-actin
beta-actin
SOX9 antibody (M00006662, Abnova)
SOX9 antibody (ab36748, Abcam)
K
H
I
SOX9 antibody (AB5535, Millipore)
sh cont
sh cont
shSOX10
sh cont
shSOX10
shSOX10
shSOX10
sh cont
shSOX10
sh cont
shSOX10
sh cont
SOX10
beta-actin
Supplementary Figure 2
