# Supplementary material for: Antagonistic Cross-Regulation between Sox9 and Sox10 Controls an Anti-tumorigenic Program in Melanoma
Source: PLoS Genet. 2015 Jan 28;11(1):e1004877. doi: 10.1371/journal.pgen.1004877 (PMC4309598; doi:10.1371/journal.pgen.1004877)
Supplement: S3 Fig — A, A schematic representation of the location of melanocytes in the hair follicular bulb. B, SOX10 expression in the hair follicular bulb. C, SOX9 expression in human basal cell carcinoma. D, Analysis of the expression of SOX10 and SOX9 in the human giant congenital naevi (patient H08 10533). Adjacent sections were stained with anti-SOX10 and anti-SOX9 antibodies. Note the positive staining for SOX9 in the hair follicle. BCC, basal cell carcinoma; GCMN, giant congenital melanocytic naevi; M, melanocytes. (PPTX) [file pgen.1004877.s003.pptx]

## Slide 1
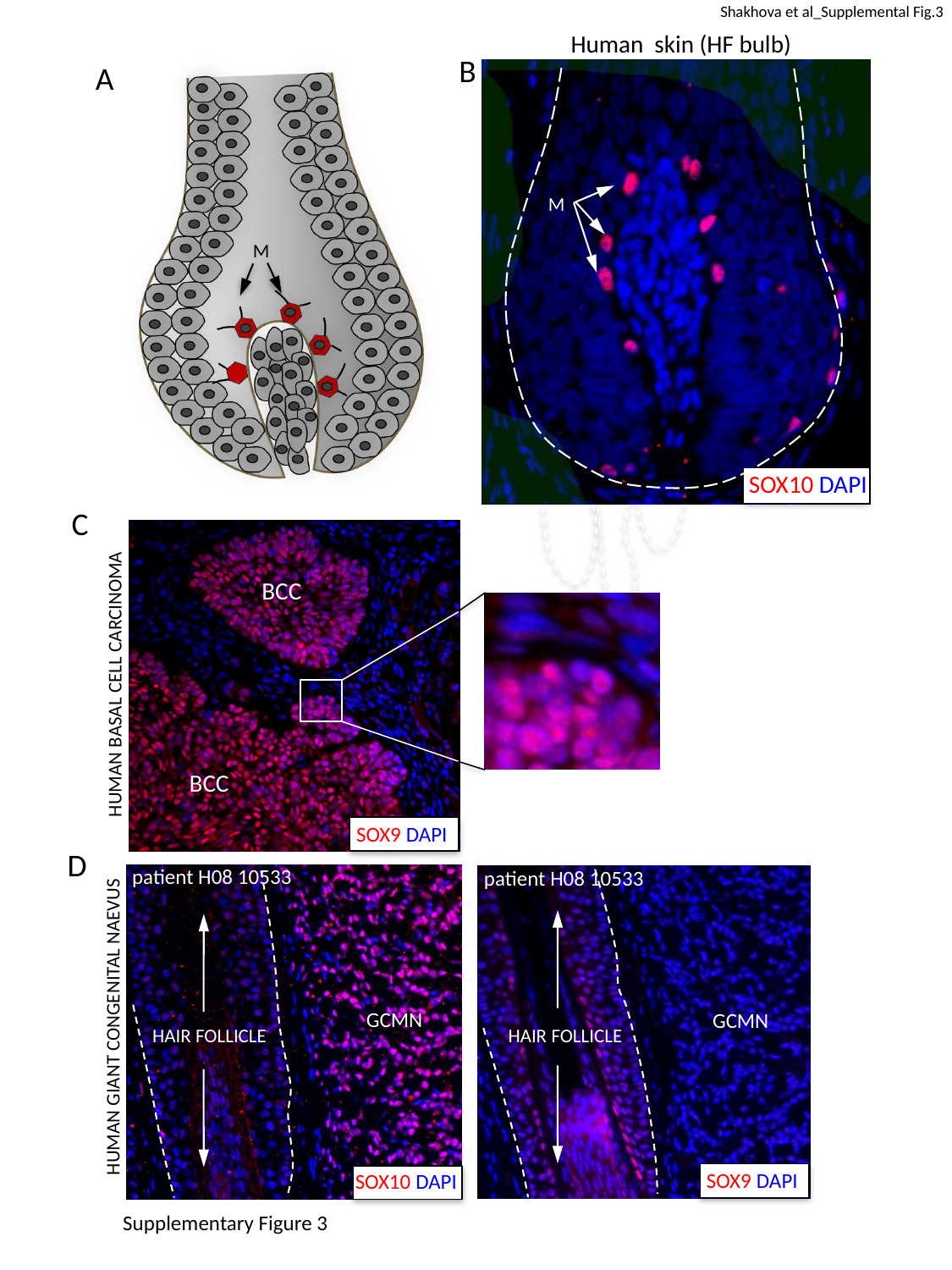

Shakhova et al_Supplemental Fig.3
Human skin (HF bulb)
B
A
epidermis
M
M
ORS
M
SOX10 DAPI
C
BCC
HUMAN BASAL CELL CARCINOMA
BCC
SOX9 DAPI
D
patient H08 10533
patient H08 10533
GCMN
GCMN
HUMAN GIANT CONGENITAL NAEVUS
HAIR FOLLICLE
HAIR FOLLICLE
SOX9 DAPI
SOX10 DAPI
Supplementary Figure 3
