# Supplementary material for: Antagonistic Cross-Regulation between Sox9 and Sox10 Controls an Anti-tumorigenic Program in Melanoma
Source: PLoS Genet. 2015 Jan 28;11(1):e1004877. doi: 10.1371/journal.pgen.1004877 (PMC4309598; doi:10.1371/journal.pgen.1004877)
Supplement: S4 Fig — A, Bright field picture (left panel) showing the pigmented melanocytes located in the hair follicular bulb. Immunostaining for SOX9 (red) demonstrating that SOX9 is expressed in the epithelial cells of the hair follicle (outer root sheath) but not in the pigmented melanocytes. B, Immunostaining for Sox9 (red) demonstrating the expression of Sox9 in the outer rooth sheath and the absence of Sox9 expression in the cells of giant congenital naevi in Tyr::NrasQ61K mouse. BF, bright field; HF, hair follicle, M, melanocytes; ORS, outer root sheath. (PPTX) [file pgen.1004877.s004.pptx]

## Slide 1
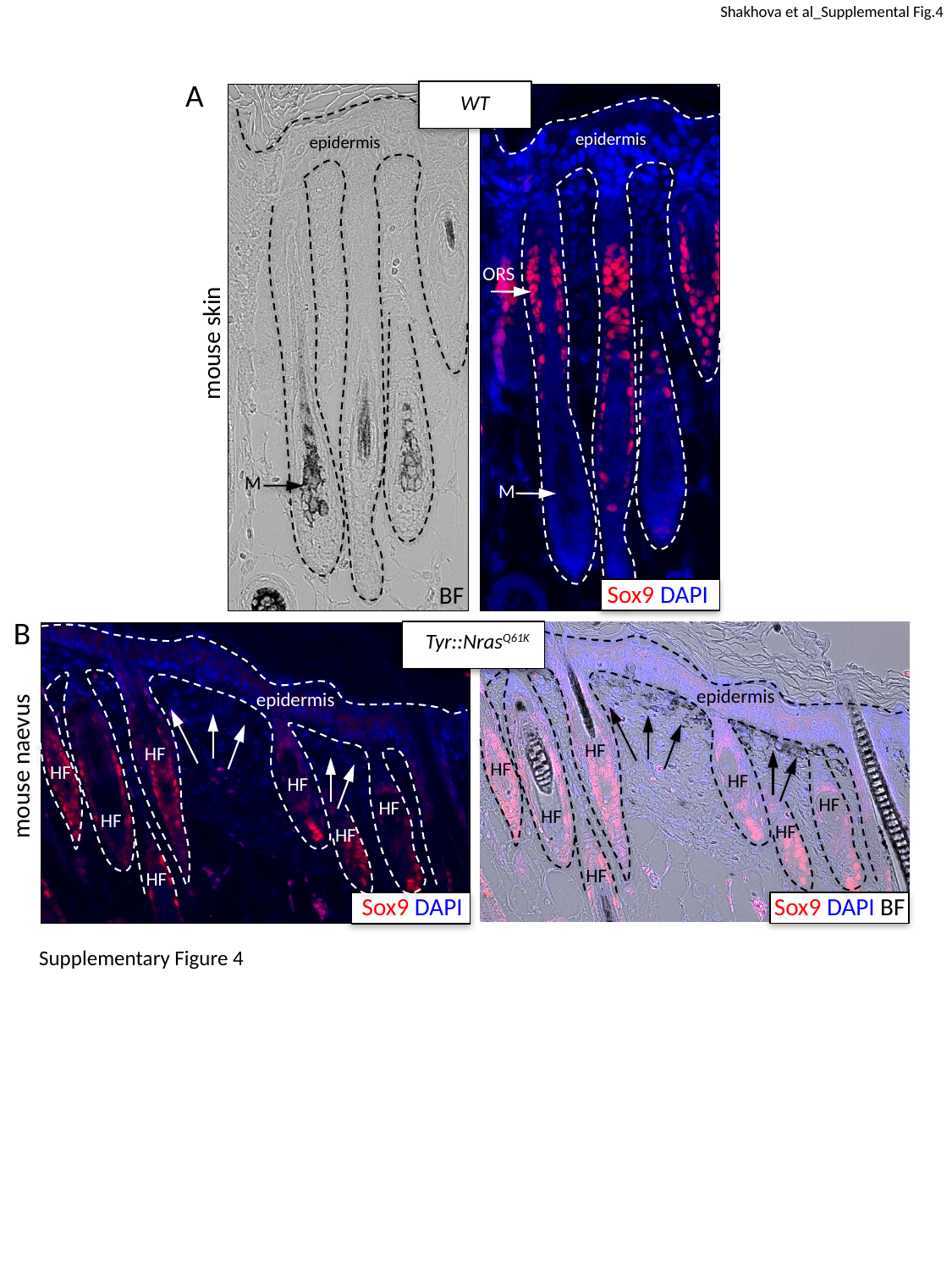

Shakhova et al_Supplemental Fig.4
A
WT
WT
epidermis
epidermis
ORS
mouse skin
M
M
Sox9 DAPI
BF
B
Tyr::NrasQ61K
epidermis
epidermis
HF
HF
mouse naevus
HF
HF
HF
HF
HF
HF
HF
HF
HF
HF
HF
HF
Sox9 DAPI BF
Sox9 DAPI
Supplementary Figure 4
