# Supplementary material for: Antagonistic Cross-Regulation between Sox9 and Sox10 Controls an Anti-tumorigenic Program in Melanoma
Source: PLoS Genet. 2015 Jan 28;11(1):e1004877. doi: 10.1371/journal.pgen.1004877 (PMC4309598; doi:10.1371/journal.pgen.1004877)
Supplement: S5 Fig — A, Western blot analysis demonstrating that SOX9 expression is upregulated upon SOX10 knockdown in human melanoma cell lines. B, FACS analysis of apoptosis in M010817 melanoma cell line. M010817 control cells, M010817 SOX10 KD cells, M010817 SOX9 OE and M010817 SOX10 KD SOX9KD cells were analyzed for the number of Annexin V-positive cells. KD, knockdown; OE, overexpression. (PPTX) [file pgen.1004877.s005.pptx]

## Slide 1
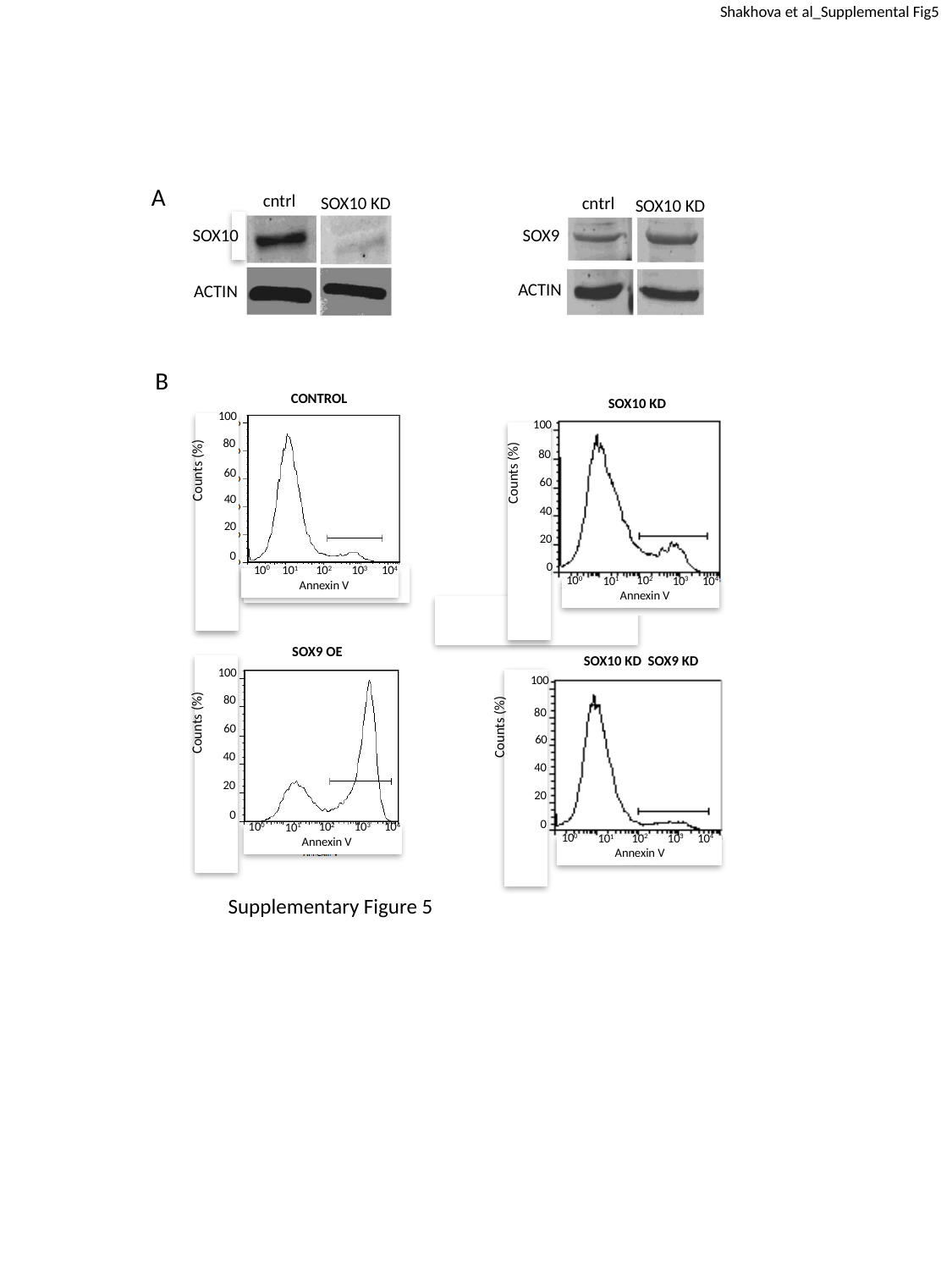

Shakhova et al_Supplemental Fig5
A
cntrl
SOX10 KD
cntrl
SOX10 KD
SOX9
SOX10
ACTIN
ACTIN
B
CONTROL
SOX10 KD
100
100
80
80
Counts (%)
Counts (%)
60
60
40
40
20
20
0
0
100
102
104
103
101
100
102
104
103
101
Annexin V
Annexin V
SOX9 OE
SOX10 KD SOX9 KD
100
100
80
80
Counts (%)
Counts (%)
60
60
40
40
20
20
0
0
100
102
104
103
101
100
102
104
103
101
Annexin V
Annexin V
Supplementary Figure 5
